# Does day 11 omission of methotrexate due to toxicity influence the outcome in myeloablative hematopoietic cell transplant? Results from a single-center retrospective cohort study

**DOI:** 10.1038/bcj.2015.70

**Published:** 2015-08-28

**Authors:** B K Hamilton, L Rybicki, H Haddad, D Abounader, M Yurch, N S Majhail, R Hanna, R Sobecks, R Dean, H Liu, B Hill, E Copelan, B Bolwell, M Kalaycio

**Affiliations:** 1 Bone Marrow Transplant Program, Department of Hematology & Oncology, Cleveland Clinic Taussig Cancer Institute, Cleveland, OH, USA; 2 Quantitative Health Sciences, Cleveland Clinic Lerner Research Institute, Cleveland, OH, USA; 3 Department of Hematology/Oncology, Staten Island University Hospital, New York, NY, USA; 4 Department of Pediatric Hematology & Oncology, Cleveland Clinic, Cleveland, OH, USA; 5 Levine Cancer Institute, Carolinas HealthCare System, Charlotte, NC, USA

Graft-versus-host disease (GVHD) remains a major cause of morbidity and mortality after allogeneic hematopoietic cell transplantation. The standard of care for GVHD prophylaxis in myeloablative transplants is methotrexate (MTX) in combination with a calcineurin inhibitor.^1^ Despite its efficacy, MTX may increase the risk of complications such as mucositis and delayed hematopoiesis and contribute to hepatic and renal toxicities.^2^ As a result, doses must often be held. Despite several studies^3, 4, 5, 6^ there remains uncertainty whether the omission of doses of MTX has a deleterious effect on relapse, GVHD and survival. We therefore reviewed our experience with MTX and omission of the day 11 dose for GVHD prophylaxis.

We identified 102 consecutive patients who underwent a myeloablative hematopoietic cell transplantation from a human leukocyte antigen-matched unrelated donor and received MTX in combination with a calcineurin inhibitor for GVHD prophylaxis from 2003 to 2011. Patient characteristics are summarized in Table 1, and transplant characteristics and definitions are as previously described.^7^

Median time to follow-up among living patients was 38 months. Seventy (69%) patients received all four doses of MTX and 32 (31%) missed the day 11 MTX. Eighty patients received MTX at a dose of 5 mg/m^2^ on days 1, 3, 6 and 11, with 24 (30%) patients having missed a dose and 25 patients having received higher-dose MTX (15 mg/m^2^ on day 1, followed by 10 mg/m^2^ on days 3, 6 and 11) after June 2009 per change in institutional practice, with eight (32%) patients having missed a dose. Severe mucositis was the primary reason for dose omission. There was no additional or alternative immunosuppressant given for patients who missed a dose of MTX. The two groups did not differ significantly in age, gender, disease, disease status, CMV status, hematopoietic cell source or preparative regimens/total body irradiation use; however, those who missed a dose of MTX were more likely to have had three or more prior chemotherapeutic regimens compared with those patients who received all four doses (40% versus 24%, respectively, *P*=0.047).

Median time to neutrophil recovery for full-dose MTX was 18 days (range 9–75) compared with 16 days (range 10–27) in the missed-dose group, *P*=0.55. Median time to platelet recovery was 25 days (range 13–51) for the full-dose group compared with 21 days (range 12–75) in the missed-dose group, *P*=0.36. The median length of stay also did not differ with a median of 34 days (range 22–98) in the full-dose MTX group compared with 35 days (range 17–109) in the missed-dose group, *P*=0.52. Mucositis, the primary reason for withholding dose of MTX, was, as expected, more severe in the missed dose group, with a mean score of 0.58±0.82, as scored by the mOMAS, compared with 0.37±0.61 in the four-dose group, although not statistically significant. Severe grade 3–4 mucositis as graded by the World Health Organization oral toxicity scale was higher in those patients who had a MTX dose withheld (62%) compared with those who received all four doses (30%), with no patients receiving all four doses of MTX reporting grade 4 mucositis.

The cumulative incidence of GVHD did not significantly differ between full- and missed-dose groups. At 6 months, there was a higher incidence of grade 2–4 acute GVHD in patients who missed a dose of MTX, 53%, versus those who received all four doses, 39% however, this was not statistically significant, *P*=0.19. There was also no difference in the incidence of grade 3–4 acute GVHD in patients missing a dose, 9% versus 20% in those receiving all four doses, *P*=0.20. Chronic GVHD developed in 35 patients, with a 2-year incidence of 30% in the full-dose group and 41% in the missed-dose group, *P*=0.34.

Cumulative incidence of relapse at 5 years was 31% in the full-dose MTX group compared with 38% in the missed-dose MTX group, *P*=0.62. The cumulative incidence of non-relapse mortality was higher (5-year incidence 58% versus 33%) (Figure 1) and overall survival was worse (5-year survival 27% versus 41%) in patients who missed a dose of MTX compared with all four doses of MTX; however, these were not statistically significant, *P*=0.06 and *P*=0.14 respectively. The cause of non-relapse death was primarily acute GVHD (*n*=7), followed by infection (*n*=5), organ toxicity/failure (*n*=4), chronic GVHD (*n*=3) and unknown (*n*=2) in patients who received all four doses of MTX. In patients who had an MTX omission, causes of non-relapse death were primarily infection (*n*=6) and organ toxicity/failure (*n*=6), followed by chronic GVHD (*n*=3) and acute GVHD (*n*=1).

In multivariable analysis, MTX omission was not associated with any acute or chronic GVHD, overall survival or relapse. However, MTX omission was a significant risk factor for non-relapse mortality (HR 1.91, 95% CI 1.01–3.62, *P*=0.048). Given association with MTX omission, additional analysis was performed to include available mOMAS mucositis data (on 98 of the 102 patients), and an mOMAS score of ⩾1.0 was found to be a significant variable for worse non-relapse mortality (HR 1.97, 95% CI 1.01–3.84, *P*=0.047), with MTX omission then becoming non-significant (*P*=0.10).

This study has several limitations, including sample size and differences and heterogeneity in our patient population. Although multivariable analysis did not identify any of these factors to be significant variables affecting incidence of GVHD or survival outcomes, there are still important factors and their interactions that we are not able to fully take into account in this analysis. We were unable to capture and analyze the potential effect of dose reductions and toxicities of cyclosporine or tacrolimus, which have also previously been shown to contribute to more severe grade III–IV GVHD,^8^ and may have further played a role in development of GVHD.

Studies have shown variable results with the omission of day 11 MTX (Supplementary Table 1).^3, 4, 5, 6^ Deeg *et al.*^3^ first noted that MTX at 10 mg/m^2^ given on days 1, 3 and 6 in combination with cyclosporine did not result in higher rates of acute GVHD compared with historically reported rates. However, a report from Nash *et al.*^8^ demonstrated that the need for a reduction in MTX to <80% of the scheduled dose was associated with an increased risk for grade IIa–IV acute GVHD. Subsequent retrospective studies have both refuted^4^ and confirmed^6^ these findings. A recent study evaluating the use of MTX in combination with tacrolimus also found no deleterious effect from omission of day 11 MTX.^5^ Another meta-analysis, evaluating individual patient data of studies using a planned four doses of MTX compared with studies using prespecified three doses on days 1, 3 and 6, found a survival advantage to receiving all four doses of MTX only for recipients of peripheral blood cells but not of bone marrow, with a significantly higher rate of relapse in recipients of bone marrow with all four doses of MTX and no differences in acute or chronic GVHD with respect to MTX dosing.^9^ In a more recent study of MTX omission, mycophenolate mofetil was substituted at the time of omission resulting in similar outcomes, although grade 2–4 acute GVHD was higher in the mycophenolate mofetil/omission arm.^10^ Interestingly, most studies demonstrating no differences in GVHD outcomes used lower doses of MTX^4, 5^ than the dose of 15 mg/m^2^ on day 1, followed by 10 mg/m^2^ day 3, 6, 11 regimen. While most institutions use this standard dosing of MTX, several studies used MTX at lower doses of 7.5 mg/m^2^ on days 1, 3, 6 and 11: 10 mg/m^2^ on day 1, followed by 7 mg/m^2^ on days 3, 6 and 11; or, as in our study, an MTX dosing of primarily 5 mg/m^2^ on days 1, 3, 6 and 11. This reduced dose schema of 5 mg/m^2^ intravenously on days 1, 3, 6 and 11 was originally developed specifically to decrease the risk of mucosal and hepatic complications, and has been widely used in combination with cyclosporine or tacrolimus as GVHD prophylaxis with historically similar outcomes as standard doses.^11^ Reduced doses of MTX, however, have never been directly compared with standard doses, and thus have never been proven equivalent.

It has been postulated that MTX omission may be a surrogate for other factors such as higher conditioning-related toxicity, which predispose patients to acute GVHD.^6^ Although it remains unclear whether a fourth dose of MTX is necessary in the absence of toxicities, omission of MTX due to mucositis or other organ toxicity appears to be a poor prognostic variable for non-relapse mortality. Severe mucositis in and of itself has previously been shown to be an independent variable contributing to inferior overall survival.^12, 13^ MTX's effect on mucositis and toxicity may also be dependent on patient and donor single-nucleotide polymorphisms of the methylenetetrahydrofolate reductase gene, which is involved in the metabolism of MTX, and is a further area of study.^14, 15^

While this study cannot definitively answer whether we can safely omit the fourth dose of MTX, it demonstrates the need for further study of MTX dosing as well as alternative regimens to MTX, given the extreme variability in dosing, toxicities and effectiveness. Until such studies are carried out, our data support the conclusion that the omission of a fourth dose of MTX in the setting of severe mucositis does not increase the risk of GVHD, but, however, may be a contributor or surrogate for worse non-relapse mortality.

## Figures and Tables

**Figure 1:**
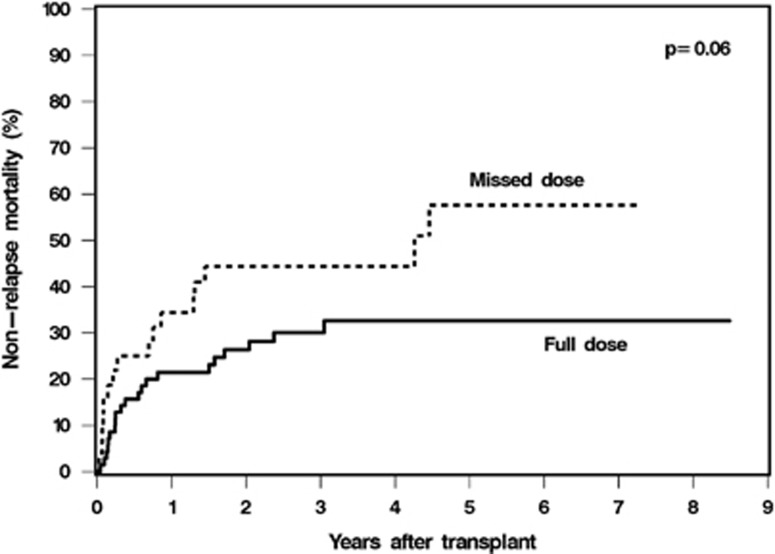
Cumulative incidence of non-relapse mortality between patients who received full-dose MTX and omission of day 11 MTX.

**Table 1:** Patient, disease and transplant characteristics (all patients received a myeloablative unrelated donor HCT)

*Variable*	*Full dose (*n=*70)*	*Missed dose (*n=*32)*	P*-value*
	N *(%)*	N *(%)*	
*Gender*			
Male	38 (54)	17 (53)	0.91
Female	32 (46)	15 (47)	
			
*Race*			
White	68 (97)	29 (91)	0.18
Other	2 (3)	3 (9)	
			
*Age at transplant, years*			
Mean±s.d.	44±12	43±12	0.74
Median (range)	47 (20–66)	47 (19–62)	
			
*Prior radiation*			
Yes	5 (7)	4 (12.5)	0.3
No	65 (93)	28 (87.5)	
			
*Comorbidity index, HCT-CI*			
Low (0)	22 (31)	15 (47)	0.21
Intermediate (1–2)	19 (27)	9 (28)	
High (3+)	29 (41)	8 (25)	
			
*Diagnosis*			
AML	29 (41)	13 (41)	0.36
MDS	19 (27)	6 (19)	
ALL	10 (14)	9 (28)	
Other	12 (17)	4 (12)	
			
*Months from diagnosis to transplant*			
Mean±s.d.	14±17	15±21	0.55
Median (range)	6 (2–81)	5 (2–106)	
			
*Disease status at transplant*			
Early	36 (51)	15 (47)	0.66
Intermediate	16 (23)	6 (19)	
Advanced	18 (26)	11 (34)	
			
*Source of hematopoietic cells*			
Bone marrow	58 (83)	27 (84)	0.85
Peripheral blood	12 (17)	5 (16)	
			
*Preparative regimen*			
Bu/Cy based	50 (71)	20 (63)	0.15
TBI/VP16	9 (13)	11 (34)	
Cy/TBI based	11 (16)	1 (3)	
			
*CMV positivity of donor/recipient*			
Yes	49 (70)	23 (72)	0.85
No	21 (30)	9 (28)	
			
*MTX dosing*			
Standard MTX	17 (24)	8 (25)	0.94
Lower dose MTX	53 (76)	24 (75)	
			
*Calcineurin inhibitor*			
Tac/MTX	63 (90)	32 (100)	0.09
CSA/MTX	7 (10)	0 (0)	

Abbreviations: ALL, acute lymphoblastic leukemia; AML, acute myelogenous leukemia; Bu, busulfan; CSA, cyclosporine; Cy, cyclophosphamide; HCT, hematopoietic cell transplantation; MDS, myelodysplastic syndrome; MTX, methotrexate; Tac, tacrolimus; TBI, total body irradiation; VP16, etoposide.
