# Supplementary material for: Does day 11 omission of methotrexate due to toxicity influence the outcome in myeloablative hematopoietic cell transplant? Results from a single-center retrospective cohort study
Source: Blood Cancer J. 2015 Aug 28;5(8):e344–. doi: 10.1038/bcj.2015.70 (PMC4558594; doi:10.1038/bcj.2015.70)
Supplement: Supplementary Table [file bcj201570x1.docx]

**Supplementary Table 1: Summary of effect of day 11 MTX omission due to toxicities**

| Study | Total N/  Missed MTX (N) | Grade 2-4 acute GVHD | Grade 3-4 acute GVHD | Non-relapse Mortality | Relapse | Overall Survival |
| --- | --- | --- | --- | --- | --- | --- |
| **Current study** | 102/(32) | NS | NS |  | NS | NS |
| Honda et al. 2013[^9^](#_ENREF_9) | 149/(22) | NS | NS | NS | NS | NS |
| Kumar et al. 2002[^10^](#_ENREF_10) | 135/(39) | NS |  | NS | **--** |  |
| Atkinson et al.  1995[^8^](#_ENREF_8) | 65/(19) | NS | **--** | NS | NS | NS |
| Nash et al.  1992[^19^](#_ENREF_19) | 446/(108) |  | **--** | **--** | **--** | **--** |

NS=no significant difference;
